# The effect of flour from the rind of the yellow passion fruit on glycemic control of people with diabetes mellitus type 2: a randomized clinical trial

**DOI:** 10.1186/s40200-017-0300-z

**Published:** 2017-04-17

**Authors:** Márcio Flávio Moura de Araújo, Vivian Saraiva Veras, Roberto Wagner Júnior Freire de Freitas, Maria do Livramento de Paula, Thiago Moura de Araújo, Lilian Raquel Alexandre Uchôa, Maria Wendiane Gueiros Gaspar, Maria da Conceição do Santos Oliveira Cunha, Maria Aparecida Alves de Oliveira Serra, Carolina Maria de Lima Carvalho, Edmara Chaves Costa, Marta Maria Coelho Damasceno

**Affiliations:** 1 Professor of University for International Integration Lusophony Afro Brazilian, CE 060, Km 51, S/N, Acarape, CEP: 62785000 Ceará Brazil; 2 Researcher from Oswaldo Cruz Foundation, Eusébio, Brazil; 3 Food Engineer, PhD in Food Sciences, Federal University of Maranhão, São Luís, Brazil; 4 Professor Federal University of Ceara, Fortaleza, Brazil

**Keywords:** Diabetes mellitus, Glycemic Index, Passiflora

## Abstract

**Background:**

The single or combined use of herbal and dietary products with medications has shown benefits in the metabolic modulation of carbohydrates, in the restoring of the function of pancreatic beta cells, and in insulin resistance. To analyze the effect of the use of flour made from the rind of the yellow passion fruit on the glycemic control of people with diabetes mellitus type 2.

**Methods:**

An open, prospective, randomized clinical trial was undertaken with 54 participants over an eight-week period. The participants from the case group were advised to ingest 12 g of the flour, three times daily; before breakfast, lunch and dinner.

**Results:**

After eight weeks of use of the flour made from the rind of the yellow passion fruit, we did not identify significant statistical differences in the values for capillary blood glucose (*p* = 0.562), fasting blood glucose (*p* = 0.268) or glycated hemoglobin (*p* = 0.229) between the study groups. In the case group, we identified an increase (29.6%–37%) of the people with normal HbA1_c_; however, this did not have statistical relevance (*p* = 0.274).

**Discussion:**

Based in our findings, we believe it is important to extend the time of exposure to the intervention and increase the rigor in the monitoring of adherence in future studies on this topic. Only in this way will we be able to make confident inferences in relation to the use of flour made from the rind of theyellow passion fruit as a therapeutic tool for glycemic and/or metabolic control in persons with DM 2.

**Conclusions:**

In the sample in question, the use of the flour made from the rind of the yellow passion fruit, over an eight-week period, did not improve the glycemic control of people with type 2 diabetes. Trial registration: U1111.1187.3616. Registered 6 September, retrospectively registered, in the Brazilian Clinical Trials Registry.

## Background

Throughout humanity’s history, herbal products and dietary supplements have been used in the treatment of diabetes type 2 (DM 2). This is the result of folk culture in the promotion of human health, which has often been looked down on. However, with the increase of diabetes and the socioeconomic and health costs associated with this, there is growing scientific interest in this issue [1–4].

The main characteristic of DM 2 is hyperglycemic, due to defects of insulin secretion, its peripheral resistance or both situations [5]. In long term this hyperglycemic framework is toxic to human body, since it stimulates the development of micro and macrovascular injuries that many times irreversible [6]. Both types have their origin in the poor glycemic control and they are responsible for morbidity and mortality rates. Diabetes complications can be divided in acute (related to eventual hyperglycemia) and chronic (related to poor metabolic control disease) [7].

The single or combined use of herbal and dietary products with medications has shown benefits in the metabolic modulation of carbohydrates, in the restoring of the function of pancreatic beta cells, and in insulin resistance. Furthermore, antioxidant properties and reduction in cardiovascular risk have been ascertained [8–10].

In virtue of this, researchers worldwide emphasize the need for studies with new natural products which employ rigorous methodological designs - the aim thus being to assess their efficacy, safety and mechanism of action [11–13]. In Brazil, one of the current proposals from the Ministry of Health is to encourage studies on decision-making in health which involve the development, validation, efficacy and consequent incorporation of technologies in the Unified Health System (SUS) [14].

One good example of this tendency is the passion fruit. Its sedative properties have been known for some time, especially when the infusion or tincture of the leaves is used [15, 16]. Recently, however, a new property has been described related to the fruit: the hypoglycemic activity of the flour produced from its rind [17–20].

Passion fruit peel is rich in soluble fibers, mainly pectin, beneficial to the human being [16, 21]. This pectin is widely used as an ingredient for pharmaceutical preparations, such as antidiarrheal and detoxifiers. In addition, it can reduce glycemic and lipid levels by forming a gel that prevents the absorption of cholesterol and glucose from the diet [22, 23].

To our knowledge, through a vast bibliographic survey, there have been only three intervention studies published on this topic. None of these, however, were characterized as randomized clinical trials [17, 20].

Brazil is a major world producer of the yellow species of passion fruit (*Passiflora edulis* f. *flavicarpa* Deg). Although the passion fruit is an organic product whose biological value is cherished in the community, its rind is not generally used. Its use in the community could create an awareness of a natural therapeutic aid in confronting DM 2. Currently, the glycemic and metabolic control of people with DM 2 is one of the main challenges in primary health care in Brazil [24, 25].

For the World Health Organization (WHO), Brazil’s herbal potential must be used in the prevention, control and treatment of health problems, especially in primary health care. In Brazil, the number of nurses who undertake studies with medicinal plants or natural products continues to remain low in relation to the country’s potential. This fact is explained by some health professionals’ lack of adhesion to multi-professional work and to knowledge of folk remedies. It is necessary for nurses to advance in studies which involve the prescribing of natural products, with the aim of legitimizing this professional practice as an interdisciplinary action of healthcare [26–28].

As a result, this study’s objective was to analyze the effect of flour made from the rind of the yellow passion fruit on the glycemic control of people with DM 2 over an eight-week period.

## Method

This is an open, prospective, randomized clinical trial, held between November 2015 and March 2016 in the city of Redenção, in the State of Ceará, Brazil.

The study population was made up of people with DM 2 who were registered and monitored in primary care in Redenção, Ceará, Brazil – numbering, at the time of the study, 9,507 people. As eligibility criteria, we established: to be a person who had had DM 2 for at least 60 days; to be aged between 18 – 65 years old, of either sex; not to have liver or kidney problems (according to data from the medical record) and not to be allergic to products derived from passion fruit. We excluded the following people from the study: people with DM 1, people who used insulin or psychotropic medications, tobacco or alcohol (all of which disrupt or control blood glucose levels), and people with mild or serious cognitive loss [29].

In the calculation of sample size, a model was used for comparing two groups by quantitative variables with pairing of the cases [30]. For this, we therefore adopted: nP – number of pairs; Zα/2 – α-error value, usually: 1.96 (5%); Zβ – β-error value, usually: 0.84 (20%); Sd – standard deviation of the difference between the pairs; $\overline{D}$– mean of the difference between the pairs.

In the above-mentioned sample calculation, we used a previous study’s mean values and values for standard deviation [17], totaling 27 pairs distributed between the intervention and control groups. As discontinuity criteria, we placed adherence inferior to 75% to this study’s intervention.

**Fig. 1:**
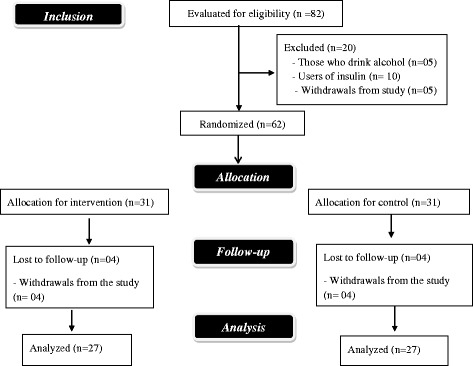
Study flow chart

For the pairing of the pairs, we took it as a rule they should have values for glycated hemoglobin (HbA1_c_) which werre as similar as possible. Following that, after the first collection of biochemical data, each member of the pairs was randomized into the intervention or control groups by means of a draw (Fig. 1).

Prior to the field research, the nurses underwent 12 h of training. The participants were interviewed regarding sociodemographic and clinical data. Following that, on a further occasion, we undertook the measuring of anthropometric and glycemic variables. These, characterized as our main outcome, were capillary blood glucose (CBG), fasting blood glucose (FBG) and glycated hemoglobin (HbA1_c_).

The collection and analysis of the biochemical specimens was undertaken by a private laboratory contracted for this purpose. The protocol for blood sample collection and capillary blood glucose, as well as values found were analyzed in accordance with the Guidelines of the Brazilian Society of Diabetes [31].

We collected 10 ml of blood, stored in two tubes of 5 ml each, with the anticoagulant sodium fluoride (for glucose determination). After the collection, the samples were processed and centrifuged at 2,200 rotations per minute (RPM) for 20 min in a digital serology centrifuge, LS3 Plus CELM®. Then, 1 ml serum and plasma samples were separated for biochemical dose determinations. In the assessment of biochemical parameters, commercial Labtest Diagnóstica S/A® kits were used, with standardized techniques, following the manufacturer’s recommendations.

We also considered, as secondary outcomes, the following variables: central adiposity index (CAI), body mass index (BMI) and waist-hip ratio (WHR). The data were collected by nurses who had received specific training, and complied with the requirements for collection and analysis made available in the literature [32, 33]. At the end of the eight-week period, the anthropometric and glycemic variables were taken again.

The participants in the intervention group were advised to ingest 12 g of the vegetable flour studied, before the three main meals (breakfast, lunch and dinner), every day, over the eight-week period. We obtained this value after reading previous papers about that subject and with the support of some professionals (food engineer, dietician and pharmacist). To this end, the members of the case group received sufficient polyurethane sachets, each containing 12 g of the product, according to analytical balance, for four weeks. They were advised to consume the product along with other foods or even in drinks.

Subsequently, we undertook home visits to deliver the remaining sachets (for the following four weeks). On these occasions, the sachets which had been distributed and taken were counted; those participants with a percentage of adherence inferior to 70% were excluded from the study (discontinuity criteria). While this was happening, the participants from the control group received the currently-used treatment from the health service involved in the study.

The yellow passion fruit peel flour was produced in the Food and Herbs laboratory. Its composition was of carbohydrates (82.7%), proteins (9.1%), ashes (5.8%) and lipids (2.2%). Each 100 g of yellow passion fruit flower equaled approximately 388 Kcal (kilocalories). It was subjected to micro and macroscopic analysis that guaranteed characteristic color, odor, taste and texture and absence of parasite, insect, rodent and general soil fragments. This analysis was guided by the quality inspection guideline of the Brazilian Association of Technical Standards (ABNT) n: 5426 [34].

We undertook comparison analysis between the groups through the use of the Student *t*-test for paired samples, in the continuous variables with normal distribution. Furthermore, we proceeded to intra-group comparison analysis, taking into account the mean values for the evaluations. In the case of asymmetric distributions, the Mann–Whitney test was used. The chi-squared test (or Fisher’s exact test) was used for comparison of the categorical variables. We made ajustments in the statistical analysis data based on BMI and WHR (confouding variables) by ANCOVA test. The statistical analyses were undertaken using the Statistical Package for the Social Sciences (SPSS, Chicago) version 11 for Windows. We considered *p* ≤ 0.05 in the calculations for inference of significant differences.

The study was approved by the Research Ethics Committee of the University for International Integration of the Afro-Brazilian Lusophony (*Universidade da Integração Internacional da Lusofonia Afro Brasileira*) in accordance with Opinion 828–534. This clinical trial was registered with the Brazilian Clinical Trials Registry (REBEC) under number U1111.1187.3616. All patients signed the informed consent form before their inclusion in the research.

## Results

We can observe that the groups were, essentially, comprised of women (intervention group 81.4% and 70.3% in the control group) who were married, retired, and who lived with family members, as shown in Table 1.

**Table 1:** Distribution of the participants, by sociodemographic characteristics. Redenção, Brazil, 2016

Variables	GROUP		*p* value
	Case	Control	
Sex	N %	N %	
Male	05 16.6	08 29.6	0.339*
Female	22 81.4	19 70.3	
Skin color			
White	08 29.6	06 22.2	0.296*
Black	02 7.4	06 22.2	
Mixed black/white	17 63	15 100	
Occupation			
Retired	20 74.0	21 81.5	0.337*
Formal employment	02 7.4	01 3.7	
Informal employment	04 14.8	01 3.7	
Housewife	01 3.7	03 1.1	
Conjugal Situation			
Married	11 40.7	14 51.8	0.864*
Single	05 18.5	04 15.8	
Widowed	08 29.6	07 26	
Separated	03 11.1	02 7.4	
Lives with -			
Family	22 81.5	18 66.6	0.449*
Partner	03 11.1	06 22.2	
Alone	02 7.4	03 11.1	

Key: *Chi-squared test

In relation to age, we can also emphasize a predominance of young-elderly in both groups: the intervention group (64.1 ± 10.9 years old) and control group (65.8 ± 10.3 years old). Sedentarism was another specific characteristic in the groups analyzed: intervention (53.3%) and control (46.6%).

We observed that the participants of the control group presented a higher monthly income (US$306.4 ± 115.5) in relation to the intervention group (US$253.2 ± 74.4) (*p* = 0.049). A substantial proportion of the subjects, from both groups, were in the social class with lower purchasing power in Brazil (D and E −74%), out of the six possible classes.

All the study participants used some form of medication for chronic health conditions. In both groups, we identified that little over half were using one or two oral antidiabetic drugs: intervention (55.5%) and control (59.2%).

From the baseline analysis, we can infer that the intervention and control groups were homogenous in relation to the outcomes of glycemic control, used in this study, as shown in Table 3.

Besides this, we did not identify significant statistical difference between groups in relation to variables BMI (*p* = 0.788), CAI (*p* = 0.233) and WHR (*p* = 0.345).

After the eight weeks of use of flour made from the rind of the yellow passion fruit, we did not identify significant statistical differences in the values for CBG (*p* = 0.562), FBG (*p* = 0.268) and HbA1_c_ (*p* = 0.229) between the groups studied (Table 2). In the intervention group, we identified an increase (29.6% – 37%) of people with normal HbA1_c_; however, this did not have statistically significant relevance (*p* = 0.274).

**Table 2:** Glycemic control of the intra-group participants after the eight week intervention. Redenção, Brazil, 2016

Variables							*p* value*
	BASELINE						
	Intervention			Control			
	M	MED	± SD	M	MED	± SD	
Capillary blood glucose (mg/dl)	176.88	155. 60	64.76	155.59	148.00	46.68	0.171*
Fasting blood glucose (mg/dl)	157.85	132.00	63.34	136.70	125.00	43.36	0.158*
Glycated hemoglobin (%)	8.73	8.05	2.37	10.5	7.50	13.96	0.522^†^
	END OF INTERVENTION						
	Intervention			Control			
	M	MED	± SD	M	MED	± SD	
Capillary blood glucose (mg/dl)	178.92	157.00	64.41	166.18	194.00	55.39	0.562*
Fasting blood glucose (mg/dl)	164.31	145.00	71.58	160.51	142.00	56.49	0.268*
Glycated hemoglobin (%)	8.40	7.90	2.35	7.70	6.90	1.80	0.229^†^

*Key: M-Mean; MED-Median; ± SD- Standard Deviation; ^†^Student *t*-test; *Mann–Whitney test

Within each group, in the means for the variables of CBG, FBG and HbA1_c_, we did not observe statistically significant differences. In the intervention group, we ascertained a slight reduction in the percentage of HbA1_c_, but this was not statistically significant over the study period (*p* = 0.608) as shown in Table 3.

**Table 3:** Glycemic control of the intra-group participants after the eight-week intervention. Redenção, Brazil, 2016

Variables							*p* value
	INTERVENTION						
	Before			After			
	M	MED	± SD	M	MED	± SD	
Capillary blood glucose (mg/dl)	176.88	156.00	64.76	178.92	157.00	62.41	0.906*
Fasting blood glucose (mg/dl)	157.85	132.00	63.34	164.31	145.00	71.54	0.726*
Glycated hemoglobin (%)	8.73	8.05	2.37	8.40	7.90	2.35	0.608^†^
	CONTROL						
	Before			After			
	M	MED	± SD	M	MED	± SD	
Capillary blood glucose (mg/dl)	155.59	148.00	46.68	166.18	153.00	55.39	0.451*
Fasting blood glucose (mg/dl)	136.70	125.00	43.36	160.51	142.00	56.49	0.088*
Glycated hemoglobin (%)	10.50	7.50	13.39	7.70	6.90	1.80	0.307^†^

*Key: M-Mean; MED-Median; ± SD- Standard Deviation; ^†^Student *t*-test; *Mann–Whitney test

Also in the intervention group, we did not observe statistically significant reductions in relation to the raw scores for the variables of CAI (*p* = 0.717), BMI (*p* = 0.958) and WHR (*p* = 0.979) in the study period.

We observed the effects among variables CBG, FBG and HbA1_c_ by ANCOVA test. It’s true that after adjusting to BMI and WHR the absence of significant statistical difference remained regarding the outcomes variables (CBG = 0.440, FBG = 0.745 and HbA1_c_ = 0.218).

## Discussion

The flour made from the rind of the yellow passion fruit (*Passiflora edulis*) is rich in pectin, a fraction of soluble fiber that has the capacity to retain water, forming viscous gels which delay gastric emptying and intestinal transit [35]. Another peculiarity of the use of this natural product is its inverse relationship with insulin resistance [36]. However, in the sample studied, its use was not efficacious in improving the glycemic control of people with DM 2 over an eight-week period. Due to some limitations of this study, it is necessary to be cautious in judging this finding.

We do not have a way of being certain that adherence to the use of the flour made from the rind of the yellow passion fruit was high (≥70%) until the end of the intervention. Given that the monitoring of the taking of the flour in person only took place during the second home visit, the rest of the contact until the final assessment was by telephone. Moreover, people with mild cognitive deficits participated in the study – and may have had some difficulty in ingesting the product.

The three previous intervention studies found in the literature observed an improvement in the glycemia, even with periods of exposure and populations which were similar, and with a lower dose of the flour (10 g, 30 g and 30 g, respectively). However, among these studies, only one adopted HbA1_C_ as an outcome, and all were based in self-controlled samples (studies of the ‘before and after’ type) [17, 18, 20].

The absence of randomization may have caused overestimation of the effect of the intervention studied in the above-mentioned publications. Furthermore, a significant proportion of the statistical techniques in the three previous studies have premises which are only achievable through the use of randomization techniques [37].

For the main specialized scientific societies, HbA1_C_ is the gold standard in assessing glycemic control. As this reflects previous glycemic control (meaning glycemia over the last 120 days) it is possible that the second measurement may reflect the prior period [38]. Even so, we ascertained reduction in this parameter (not significant) in only eight weeks in this sample.

The importance of including foods or plants which promote an improvement in glucose tolerance, in the diets of people with DM, has been studied in primary health care in Brazil [39–42]. This fact can be explained by the richness of Brazil’s natural heritage and by the need to seek tools of care in the cultures of the different regions of Brazil? (Brazil’s National Health Promotion Policy). In spite of this, outside Brazil, there continues to be a great need to list new evidence and/or information in order to definitively officialize the use of natural products in the traditional clinical care of the person with DM [11, 12, 43].

It is incorrect to think that all natural and/or phytotherapy products are totally efficacious and/or safe in the control of DM [1]. Indeed, the American Diabetes Association (ADA) and/or the SBD do not have any specific guidelines regarding the management of these alternative products or therapies. There is also an absence of robust evidence demonstrating the efficacy of dietary supplements in the management of diabetes. This is principally the result of the lack of standardization of these formulas, and of the studies’ small samples and methodological flaws [1, 44]. The ADA accepts these products’ use so long as they are recommended and/or used under the supervision of a health professional who must assess the safety and efficacy of these products prior to indicating them to the patients [44–46].

Based in our findings, we believe it is important to extend the time of exposure to the intervention and increase the rigor in the monitoring of adherence in future studies on this topic. Only in this way will we be able to make confident inferences in relation to the use of flour made from the rind of the yellow passion fruit as a therapeutic tool for glycemic and/or metabolic control in persons with DM 2. In addition, it is important to extend the study of the effects of this vegetable product among people with other chronic health conditions, such as metabolic syndrome, obesity and hypertension.

## Conclusion

In the sample in question, the use of flour made from the rind of the yellow passion fruit, over an eight-week period, did not improve glycemic control of people with type 2 diabetes.

## Abbreviations

ABNT: Brazilian Association of Technical Standards
ADA: American Diabetes Association
BMI: Body mass index
CAI: Central adiposity index
CBG: Capillary blood glucose
DM 2: Diabetes type 2
FBG: Fasting blood glucose
HbA1c: Glycated hemoglobin
REBEC: Brazilian Clinical Trials Registry
SBD: Brazilian Society of Diabetes
SPSS: Statistical Package for the Social Sciences
SUS: Unified Health System
WHO: World Health Organization
WHR: Waist-hip ratio
